# Treatments for pathogen infection rescued 2 patients with renal artery rupture after kidney transplantation: A case report

**DOI:** 10.1097/MD.0000000000039124

**Published:** 2024-08-02

**Authors:** Xin Lian, Si Liu, Jie Zhang, Lei Pang, Xuemei Yi, Gang Wang

**Affiliations:** a Department of Urology, The First Hospital of Jilin University, Changchun, China; b Department of Anesthesia, The First Hospital of Jilin University, Changchun, China; c Department of Second Operation Room, The First Hospital of Jilin University, Changchun, China.

**Keywords:** bacterial infection, case report, fungal infection, renal artery rupture, renal transplantation

## Abstract

**Rationale:**

Renal artery rupture due to allograft infection, especially by fungi, is a serious clinical complication that can occur after kidney transplantation, and may lead to graft loss and death.

**Patient concerns:**

Two kidney recipients from China who developed renal artery rupture at our hospital on 5 days (47-year-old female) and 45 days (39-year-old male) after surgery.

**Diagnoses:**

The male had immunoglobulin A nephropathy as a primary disease, and experienced a postoperative attack of vascular rejection and mixed infection by *Mucor* and bacteria. The female had chronic glomerulonephritis as a primary disease, and experienced renal artery rupture near the anastomosis site with infection by fungi and other pathogens.

**Interventions:**

The male received resection of the implanted kidney and antibiotic therapy with intravenous vancomycin (0.5 g, 2 days) and amphotericin B (530 mg in 33 days). The female received replacing the segment of renal arterial and internal iliac artery by saphenous vein, as well as antibiotic therapy with amphotericin B (320 mg in 8 days).

**Outcomes:**

The male was recovered and received a second transplantation, while the female was discharged on postoperative day 19.

**Lessons:**

In both patients, prompt surgery and aggressive treatment with an antifungal drug (amphotericin B) and antidrugs led to successful rescue.

## 1. Introduction

Kidney transplantation is a life-saving renal replacement therapy, but various postoperative complications can occur, such as delayed graft function, hypertension, proteinuria, and adverse effects from the various medications. One of the most serious complications is renal artery rupture due to infection by a viral, fungal, or bacterial pathogen, because these infections are associated with increased morbidity and mortality.^[1]^ These infections can manifest as a hematogenous or local spread of bacteria or fungi to the renal arterial vessel due to donor-to-host transmission, but are more commonly due to contamination during organ procurement.^[2]^ posttransplant infections are generally less common in high-volume transplant centers. There are approximately 250 kidney transplant surgeries per year at our center, and these kidneys are from about 200 unrelated individuals and 50 relatives. After 1 year, the patient survival rate exceeds 99% and the graft survival rate is about 90%.

Although infection is a common and well-known postsurgical complication, it remains a major cause of death in renal transplant recipients. We describe 2 patients who were first-time recipients of renal transplantation from living relatives. One patient had immunoglobulin A (IgA) nephropathy and the other patient had chronic glomerulonephritis. Each patient developed renal artery rupture with infection. We administered surgical interventions, antifungal and antibacterial therapies, and immunomodulators to rescue these 2 patients. Herein, we summarize the main clinical features and describe the clinical outcomes of these 2 patients, and then discuss the main issues that should be considered for the effective treatment of infectious complications after renal transplantation.

## 2. Case report

### 2.1. Transplantation related information

The first case was a 39-year-old male who had immunoglobulin A nephropathy as a primary disease, and received a first-time renal transplant from his older sister in January 2007 (Table 1). The preoperative tests of human antigens and the panel of reactive antibodies were negative, and serological analysis indicated a total HLA mismatch level of 0 to 3. Cultures of blood and urine from the donor and the kidney preservation medium showed no abnormalities. The allograft was performed using end-to-end anastomosis of the donor renal artery with the internal iliac artery, and end-to-side anastomosis of the donor renal vein with the external iliac vein. The immunosuppressive regimen during the transplantation consisted of 200 mg of antihuman lymphocyte globulin and 500 mg of methylprednisolone (MP) (Table 2).

**Table 1:** Characteristics of renal recipients, type of anastomosis for transplantation, and culture results from donors before transplantation.

	Case 1	Case 2
Recipient		
Gender	Male	Female
Age, yrs	39	47
Primary disease	IgA nephropathy	Chronic glomerulonephritis
Type of anastomosis	End-to-end	End-to-end
Donor		
Relationship	Older sister	Younger sister
Blood culture	Negative	Negative
Urine culture	Negative	Negative
Sputum culture	Not applicable	Not applicable
Preservation solution culture	Negative	Negative

**Table 2:** Immunosuppressive and antibiotic regimens.

	Case 1	Case 2
IS regimen before surgery		
Basiliximab	No	No
Antihuman T lymphocyte globulin	No	No
IS regimen during surgery		
Basiliximab	No	No
Antihuman T lymphocyte globulin	200 mg/d	200 mg/d
Methylprednisolone	500 mg/d	500 mg/d
IS regimen after surgery		
Basiliximab	No	No
Antihuman T lymphocyte globulin	No	No
Methylprednisolone	500 mg/d, 2 d	500 mg/d, 2 d
Tacrolimus (FK506)	–	–
Prednisone	–	
Mycophenolate mofetil	3 g/d	3 g/d
Antibiotic prophylaxis after surgery	2G cephalosporin	2G cephalosporin

2G = second-generation, IS = immunosuppressive.

The second case was a 47-year-old female who had a history of chronic glomerulonephritis and received a first-time renal transplant from her younger sister in December, 2019 (Table 1). The preoperative evaluations, renal transplant procedure, and immunosuppressive regimen were the same as in case 1.

### 2.2. Postoperative course and treatment in case 1

**Figure 1.**
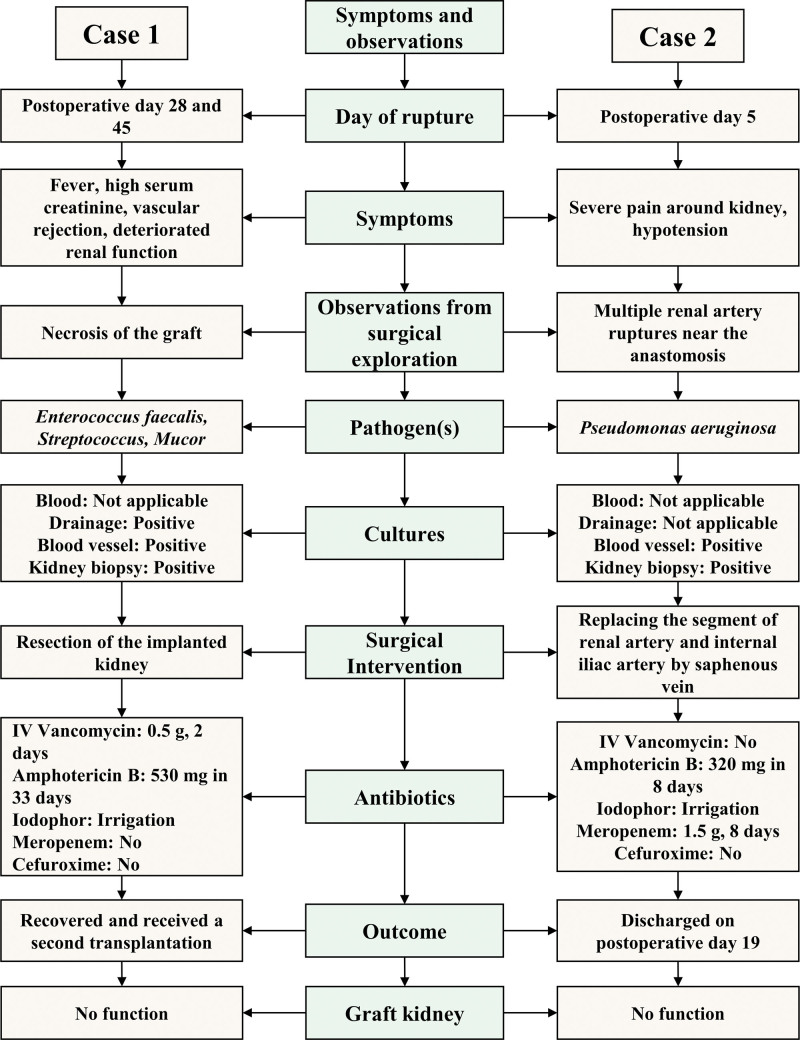
Symptoms and observations from surgical exploration, culture results, and interventions after renal artery rupture.

The postoperative condition of case 1 was uneventful for several days, and he received MP (500 mg/day) for 2 days and a second-generation of cephalosporin (3 g/day) (Table 2). The patient’s kidney function was restored on postoperative day 4, but he started to develop a fever with a slow increase in serum creatinine (SCr). Pathological analysis confirmed mild to moderate vascular rejection (Fig. 1). The family refused treatment with a monoclonal antibody, and the 2 days of MP treatment seemed ineffective. His kidney function deteriorated gradually, and the implanted kidney was therefore resected on postoperative day 28. During the emergency exploratory procedure, we noticed mild necrosis of the allograft, without obvious fresh blood clots, and rupture of the renal artery. Subsequent cultures led to identification of *Enterococcus faecalis* in the necrotic tissues, and *Streptococcus* and *Mucor* in the drainage fluid (Fig. 1).

On postoperative day 45, another episode of septic shock occurred during dialysis, and this was accompanied by a sudden increase of drainage up to 1000 mL. An emergency reexploration revealed fluid around multiple small ruptures on part of right common iliac artery and the right external iliac artery. Bacterial and fungal infections were subsequently confirmed by cultures. Because the arterial wall was fragile and it was difficult to stop the bleeding, the affected part of artery was resected and femoro-femoral bypass grafting was then performed (Fig. 1). *Staphylococcus epidermidis* was recovered from cultures of the resected necrotic tissues and blood vessels, and intravenous vancomycin (0.5 g/day, 12 days) and amphotericin B (530 mg over 33 days; gradually increased from 3 to 25 mg/day, and then decreased to 10 mg/day) in combination with local iodine irrigation were administered until the drainage cultures tested negative. The patient received a second renal graft 12 years after the initial transplant, and his kidney function gradually recovered.

### 2.3. Postoperative course and treatment in case 2

The immediate postoperative condition of case 2 was also uneventful, and she also received MP (500 mg/day) for 2 days and a second-generation cephalosporin (2 g/day) (Table 2). However, she developed severe pain around the graft sites and hypotension (lowest BP: 51/38 mm Hg) on postoperative day 5 (Fig. 1). Emergency exploration revealed a 2 mm rupture of the renal artery that was about 2 mm distal to the anastomosis between the renal artery and the recipient internal iliac artery. However, after suture repair, we observed a 3 × 3 mm dark area on the renal artery close to the anastomosis, which appeared to be fungal infection. The infected segments (internal iliac artery and renal artery about 1.5 to 2 cm distal to anastomosis) were resected and replaced by a saphenous vein. In this procedure, the donor kidney was perfused by preservation fluid, followed by anastomosis of the saphenous vein to the renal artery and internal iliac artery. A culture from the resected vessels showed *Pseudomonas aeruginosa*. We therefore administered meropenem (1.5 g/day) and amphotericin B (320 mg over 8 days) until postoperative day 8, at which time there were negative results from the drainage cultures. The patient’s kidney function improved gradually thereafter.

## 3. Discussion

A renal transplant recipient can develop an infection due to transmission from the donor, exogenous contamination during graft handling and implantation, and endogenous factors within the recipient. However, our preoperative evaluations indicated that the risk of donor-derived infection was minimal in our 2 patients.

Mucormycosis is a rare but highly lethal fungal infection, and the mortality rate can range from 40% to 100%.^[3,4]^ In renal medicine, mucormycosis was previously reported in immunosuppressed patients following transplantation and in patients receiving desferrioxamine, a chelating agent used to treat iron-overload due to hemodialysis.^[5–7]^ The factors responsible for mucormycosis in our case 1 are unclear, but infection through the incision at the time of transplantation is a probable portal of entry for fungal spores. We were able to identify *Mucor* in this patient’s drainage fluid on postoperative day 28. We attribute the survival of our patient to the prompt surgery and administration of liposomal amphotericin B. Our use of a second-generation cephalosporin may also have helped to minimize interference from bacteria. Consistent with our findings, a previous case report described a patient with IgA nephropathy who was successfully treated with liposomal amphotericin B for mucormycosis after renal transplantation.^[8]^ Thus, although mucormycosis is rare, it appears to be a serious complication for renal transplant recipients. Early diagnosis and prompt institution of surgical and antifungal therapy appear to be key for these patients.^[6,8]^

In additional to fungal infection, previous studies reported that artery ruptures near the anastomosis site were associated with bacterial infections and surgical complications.^[2,9,10]^ For example, a review of the literature reported that 73 of 87 renal transplant recipients (83.9%) experienced vascular complications at the anastomosis site^[11]^; among these 87 patients, 69 (79.3%) had pseudoaneurysm, 18 (20.7%) had arterial rupture, and 54 (62.1%) had evidence of infective pathogens.^[11]^ Orlando et al^[12]^ reported the death of renal transplant recipient due to an arterial anastomotic rupture caused by *P. aeruginosa.* In contrast, our case 2 had a *P. aeruginosa* infection, but recovered because of the rapid surgical intervention and effective therapy with meropenem and amphotericin B. In addition, early nephrectomy is considered a better approach than vascular repair for the rescue these patients,^[13,14]^ because the later approach is associated with a high recurrence of massive bleeding.^[13–15]^ Notably, Matignon et al^[16]^ demonstrated favorable outcomes of 8 patients who received renal transplantation with prophylactic antifungal treatment due to *Candida* contamination of the preservation fluid. Another study of patients who received renal transplants from deceased donors found that a 2-week preemptive antifungal prophylaxis prevented vascular complications.^[17]^ Thus, the preservation of renal function in our case 2 may be attributable to the early antifungal treatment, which slowed the growth of fungi and decreased the detrimental effects on renal blood vessels.

Aggressive surgical reconstruction can preserve the renal graft in a recipient who has vascular complications, but recovery of the graft remains a challenge. The routine use of prophylactic antifungal treatment in renal recipients may lead to more favorable outcomes, but evidence is needed for confirmation. The prompt detection of an infected artery or vessel rupture after renal transplantation, followed by immediate surgical treatment and administration of antifungal medications are essential for improving patient survival. We suggest that future developments should focus on improving early diagnosis and implementing more effective and less toxic antifungal prophylactic strategies.

## Author contributions

**Conceptualization:** Xin Lian, Si Liu, Jie Zhang.

**Data curation:** Xin Lian, Si Liu, Jie Zhang.

**Visualization:** Xin Lian, Si Liu, Jie Zhang, Lei Pang, Xuemei Yi.

**Writing – original draft:** Xin Lian, Si Liu, Jie Zhang, Lei Pang, Xuemei Yi.

**Writing – review & editing:** Lei Pang, Gang Wang.

## References

[1] Nasr SH Radhakrishnan J D’Agati VD. Bacterial infection-related glomerulonephritis in adults. Kidney Int. 2013;83:792–803. 23302723 10.1038/ki.2012.407

[2] Vidne BA Leapman SB Butt KM. Vascular complications in human renal transplantation. Surgery. 1976;79:77–81. 1108262

[3] Rabkin JM Oroloff SL Corless CL. Association of fungal infection and increased mortality in liver transplant recipients. Am J Surg. 2000;179:426–30. 10930495 10.1016/s0002-9610(00)00366-4

[4] Patel R Portela D Badley AD. Risk factors of invasive Candida and non-Candida fungal infections after liver transplantation. Transplantation. 1996;62:926–34. 8878386 10.1097/00007890-199610150-00010

[5] Boelaert JR van Roost GF Vergauwe PL. The role of desferrioxamine in dialysis-associated mucormycosis: report of three cases and review of the literature. Clin Nephrol. 1988;29:261–6. 3293856

[6] Mitwalli A Malik GH al-Wakeel J. Mucormycosis of the graft in a renal transplant recipient. Nephrol Dial Transplant. 1994;9:718–20. 7970104 10.1093/ndt/9.6.718

[7] Andrews PA Abbs IA Koffman CG. Mucormycosis in transplant recipients: possible case–case transmission and potentiation by cytomegalovirus. Nephrol Dial Transplant. 1994;9:1194–6. 7800228 10.1093/ndt/9.8.1194

[8] Serna JH Wanger A Dosekun AK. Successful treatment of mucormycosis peritonitis with liposomal amphotericin B in a patient on long-term peritoneal dialysis. Am J Kidney Dis. 2003;42:E14–17. 12955706 10.1016/s0272-6386(03)00797-2

[9] Goldman MH Tilney NL Vineyard GC. A twenty year survey of arterial complications of renal transplantation. Surg Gynecol Obstet. 1975;141:758–60. 1105838

[10] Russo VR Marks C. Renal transplantation: an analysis of operative complications. Am Surg. 1976;42:153–9. 769618

[11] Lin YH Liao CH Jiang BJ. Early renal arterial rupture and arterial pseudoaneurysm in graft kidneys from the same deceased donor. Ci Ji Yi Xue Za Zhi. 2018;30:250–4. 30305791 10.4103/tcmj.tcmj_180_17 PMC6172893

[12] Orlando G Di Cocco P Gravante G. Fatal hemorrhage in two renal graft recipients with multi-drug resistant *Pseudomonas aeruginosa* infection. Transpl Infect Dis. 2009;11:442–7. 19508700 10.1111/j.1399-3062.2009.00412.x

[13] Zhu X Liu H Wang W. Two cases of transplant renal artery thrombosis and spontaneous rupture caused by mucormycosis. Transpl Infect Dis. 2015;17:442–8. 25846151 10.1111/tid.12387

[14] Mai H Champion L Ouali N. *Candida albicans* arteritis transmitted by conservative liquid after renal transplantation: a report of four cases and review of the literature. Transplantation. 2006;82:1163–7. 17102767 10.1097/01.tp.0000239188.27153.23

[15] Santangelo ML Bracale UM Carlomagno N. Kidney transplantation and large anastomotic pseudoaneurysm. Transplant management considerations. Ann Ital Chir. 2013;84:275–9. 23135415

[16] Matignon M Botterel F Audard V. Outcome of renal transplantation in eight patients with Candida sp. contamination of preservation fluid. Am J Transplant. 2008;8:697–700. 18294166 10.1111/j.1600-6143.2007.02112.x

[17] Zhao DD Huang ZY Hong LQ. Massive hemorrhage caused by fungal infections after donation-after-cardiac-death kidney transplantation: clinical features, prevention and treatment experience. Zhonghua Yi Xue Za Zhi. 2016;96:1570–2. 27266684 10.3760/cma.j.issn.0376-2491.2016.20.005

